# Walking Ability Associated with Executive Dysfunction in Patients with Stroke: A Cross-Sectional Study

**DOI:** 10.3390/brainsci13040627

**Published:** 2023-04-06

**Authors:** Katsuya Sakai, Yuichiro Hosoi, Yusuke Harada

**Affiliations:** 1 Department of Physical Therapy, Faculty of Health Sciences, Tokyo Metropolitan University, Tokyo 116-8551, Japan; 2 Department of Rehabilitation of Medicine, Keio University School of Medicine, Tokyo 160-8582, Japan; s1455105@sc.sozo.ac.jp; 3 Department of Sports Health Sciences, Ritsumeikan University, Kusatsu 525-3760, Japan; 4 Department of Rehabilitation, Reiwa Rehabilitation Hospital, Chiba 260-0026, Japan; harada1111.0917@gmail.com; 5 Graduate School of Human Health Sciences, Tokyo Metropolitan University, Tokyo 116-8551, Japan

**Keywords:** executive dysfunction, trail making test, walking, stroke, cluster analysis

## Abstract

Previous studies have shown an association between executive dysfunction and walking ability. However, it remains unclear whether the degree of executive dysfunction is associated with differences in walking ability in patients with stroke. The aim of this study was to investigate whether there are differences in walking ability according to executive dysfunction in patients with stroke. A total of 51 patients with stroke were enrolled in this study. Executive function was measured using the Trail Making Test (TMT) Part B, and walking ability was assessed using the 10 m walk test and the Timed Up and Go Test (TUGT). Cluster analysis was performed using the TMT Part B and compared within each cluster. TMT Part B was categorized into three groups (cluster 1: *n* = 20, cluster 2: *n* = 24, and cluster 3: *n* = 7). Cluster 1 was significantly better than clusters 2 and 3, and cluster 2 was significantly better than cluster 3. The 10 m walk time and TUGT of cluster 1 were significantly better than those of cluster 3. However, the 10 m walk time and TUGT of clusters 1 and 2 did not differ significantly. In conclusion, these findings may indicate differences in walking ability according to executive dysfunction.

## 1. Introduction

Executive function is a higher brain function that involves various factors, including decision making, risk taking, planning, inhibitory control, working memory, and cognitive flexibility (speed, error processing, and attention) [1]. Executive dysfunction refers to difficulty with these functions. Previous studies have reported that approximately 47–75% of patients with stroke exhibit executive dysfunction [1,2,3,4,5], which considerably impacts the activities of daily living (ADL) and their reintegration into society [6,7]. Lipskaya-Velikovsky et al. investigated whether executive dysfunction is linked to ADL function in patients with stroke [6], and found a correlation between executive dysfunction and ADL function. In addition, Ownsworth et al. investigated whether the return to work is affected by executive dysfunction in patients with stroke, and observed that patients with stroke who had executive dysfunction were less likely to return to work than patients with stroke who did not have executive dysfunction [7].

The prefrontal cortex (frontal lobe), basal ganglia, and cerebellum are the brain regions mainly associated with executive function [1,8]. These regions, which are connected by white matter fibers [8], activate not only motor control but also cognitive function, including executive function. Therefore, direct or indirect damage (i.e., stroke) to these areas may lead to executive dysfunction [1,8,9]. Specifically, patients with stroke due to frontal lobe lesions show poorer executive function than those with stroke due to lesions in other regions [9].

Executive dysfunction in patients with stroke can be assessed using the Trail Making Test (TMT), Behavioural Assessment of Dysexcutive Syndrome (BADS), Frontal Assessment Battery (FAB), and Stroop Color and Word Test [10,11,12,13]. The TMT has widely been applied to evaluate executive dysfunction owing to its ability to quickly assess patients with stroke [11,14,15]. The TMT is categorized into two parts (parts A and B). The TMT Part A involves connecting the numbers 1–25 in order as quickly as possible, whereas the TMT Part B involves connecting the numbers 1–13 and letters in order, alternating between numbers and letters. A study has shown that the TMT stimulates large-scale brain networks, including the prefrontal cortex and parietal region [16]. Moreover, studies have established that the TMT Part B reflects executive function (i.e., attention, memory, sequencing, decision making, automatic thinking, set shifting, and cognitive flexibility) [14,17,18]. The TMT Part B has the limitation that it can only assess limited aspects of executive dysfunction compared with the BADS and FAB. However, this test possesses the advantage of being able to assess executive dysfunction intuitively, which makes it an easy assessment to be performed in clinical practice.

Previous studies have demonstrated a relationship between executive dysfunction and physical function in patients with stroke [5,15,19]. Specifically, executive dysfunction has been shown to be associated with balance ability in patients with stroke [5]. Hayes et al. used BADS to investigate executive dysfunction and balance ability in patients with stroke [5], and showed that executive dysfunction was linked to poor balance ability. Liu-Ambrose et al. utilized the Stroop test to examine executive dysfunction and determine balance ability in community-dwelling older adults after a mild stroke [19], and found that executive dysfunction was associated with balance ability. Furthermore, previous reports have shown that executive dysfunction was associated with walking ability [15,19]. Hayes et al. investigated whether executive dysfunction determined using various assessments was associated with the 10 m walking test in patients with stroke [15]. They reported an association between the 10 m walking time and executive dysfunction in patients with stroke. However, it is unclear whether differences exist between the degree of executive dysfunction and walking ability in patients with stroke. Therefore, this study aimed to investigate whether there are differences in walking ability according to executive dysfunction in patients with stroke.

## 2. Material and Methods

### 2.1. Participants

A total of 51 patients with stroke were enrolled (average age, 67.1 ± 13.3 years; body mass index, 23.1 ± 3.20 kg/m^2^; 36 men; average time since stroke, 66.7 ± 46.0 days; infraction, 30; left hemiplegia, 36 patients). This cross-sectional study was performed at two rehabilitation hospital units between October 2021 and June 2022. The inclusion criteria were as follows: (1) patients with first-time stroke, (2) ability to walk using aids, (3) age > 20 years, (4) absence of lower limb orthopedic disease, and (5) presence of hemiplegia. The exclusion criteria were as follows: (1) diagnosis of dementia and (2) diagnosis of higher brain dysfunction (e.g., unilateral spatial neglect, aphasia, or apraxia). The participants received an explanation regarding the purpose of this study, after which written informed consent was obtained prior to study initiation. This study was approved by the ethics committee of the Ukai Rehabilitation Hospital and Reiwa Rehabilitation Hospital (approval number: 20210907, 00001), publicly registered in UMIN Clinical Trials Registry (UMIN-CTR) (trial registration ID: UMIN000048479), and complied with the ethical standards established in the 1964 Declaration of Helsinki.

### 2.2. Assessments

Executive and cognitive function was assessed using the TMT Parts A and B and the Mini-Mental State Examination (MMSE). Walking ability was assessed using the maximum 10 m walking speed test and the Timed Up and Go Test (TUGT). Other physical functions were assessed using Brunnstrom Recovery Stage (BRS), sensory part of the Stroke Impairment Assessment Set (SIAS), Berg Balance Scale (BBS), and Functional Independence Measure (FIM).

#### 2.2.1. TMT

The TMT Part A reflects motor speed and attention, whereas the TMT Part B has often been used to assess executive function [14,17,18]. Therefore, this study used the TMT Part B as it reflects executive function. For the TMT Part A, participants connected the circled numbers (from 1 to 25) in sequence as fast as possible [11,14]. For the TMT Part B, they connected numbers and letters alternatingly in sequence as fast as possible [11,14].

#### 2.2.2. MMSE

The MMSE was used to assess cognitive function, with a total score of ≥30 points indicating better cognitive function [20]. The scores were interpreted as follows: 0–10 = severe cognitive impairment, 11–20 = moderate cognitive impairment, 21–29 = mild cognitive impairment, and 30 = normal cognition [20].

#### 2.2.3. Maximum 10 m Walking Test

The maximum 10 m walking test was performed on a 16 m straight walking pathway. The test was found to be reliable among patients with stroke [21]. The participants were instructed to maintain their maximum walking speed along the entire pathway. Their maximum 10 m walking speed was measured over the central 10 m of the pathway, excluding the distance before and after 3 m, using a stopwatch. The participants walked with a cane or orthotic device, as they would in their daily lives. The maximum 10 m walking test was conducted twice, and the average value was used for the analysis.

#### 2.2.4. TUGT

The TUGT was performed twice at a comfortable speed. The participants were instructed to stand up from the chair, walk to a cone 3 m away from the chair, walk around it toward their nonparalytic side, return to the chair, and sit down [22]. The proctor instructed the participant to stand up from the chair at the ‘ready to go’ cue and measured the time using a stopwatch. The participants were allowed to use walking aids and orthotics that they normally use in their daily life. Thereafter, the average values for TUGT were calculated.

#### 2.2.5. BRS

The BRS is a 6-point scale used to assess the degree of motor paralysis as well as upper limb function, finger function, and lower limb function [23], with higher scores on this index (score range, 1–6) indicating better motor function.

#### 2.2.6. SIAS

The SIAS is a comprehensive tool for assessing motor and sensory function in patients with stroke [24]. The SIAS, which comprises 22 items, classifies functional disorders into nine types. This study only used the item on sensory function (tactile sense and position sense). Sensory function is rated on a 4-point scale (score range, 0–3). The better the sensory function, the higher the points.

#### 2.2.7. BBS

The BBS was used to assess balance [25]. This tool comprises 14 items, each rated on a 5-point scale (0–4). The BBS has a total score of 56 points, with higher scores indicating better balance.

#### 2.2.8. FIM

The FIM, which measures the ability to perform ADL [26], comprises a total of 18 items: 13 motor items (FIM motor) and 5 cognitive items (FIM cognitive) [27]. Each item is rated using a 6-point scale (score range, 1–7), with 91, 35, and 126 being the highest scores for FIM motor, FIM cognitive, and FIM total, respectively. Higher scores indicate better function in ADL.

### 2.3. Statistical Analysis

The Shapiro–Wilk test was used to determine the distribution of the data. Cluster analysis (Ward’s method) was performed using the TMT Part B. Either one-way analysis of variance or Kruskal–Wallis test was used to determine the differences in values among the three groups. The Bonferroni method was used to perform multiple comparisons. The Chi-square test was used to determine differences in sex, type of stroke, and paretic side. Spearman’s rank correlation analysis was performed to evaluate the relationships between the TMT Part B and various variables (the TMT Part A, MMSE, maximum 10 m walking time, TUGT, BRS, sensory part of SIAS, BBS, and FIM). Statistical analysis was performed using SPSS 28.0 (SPSS Inc., Chicago, IL, USA), with *p* < 0.05 indicating statistical significance.

## 3. Results

A total of 51 patients with stroke were enrolled (average age, 67.1 ± 13.3 years; body mass index, 23.1 ± 3.20 kg/m^2^; 36 men; average time since stroke, 66.7 ± 46.0 days; infraction, 30; and left hemiplegia, 36 patients) (Table 1).

Three clusters were identified with the results of cluster analysis using the TMT Part B (cluster 1: the mild executive function group, *n* = 20; cluster 2: the moderate executive function group, *n* = 24; cluster 3: the severe executive function group, *n* = 7; Table 1). No significant differences in basic attributes were observed among the three groups (*p* > 0.05, Table 1). However, age and time since stroke were significantly different (age: F = 5.12, *p* = 0.010, time since stroke: H = 6.25, *p* = 0.044). The results of multiple comparisons using the Bonferroni method showed that cluster 1 was significantly different from cluster 3 (age: *p* = 0.020, time since stroke: *p* = 0.046, Table 1).

The results for physical and cognitive functions are shown in Table 2. There were significant differences in the TMT Part B among the three clusters (H = 41.68, *p* < 0.001). The results of multiple comparisons using the Bonferroni method showed that the TMT Part B score of cluster 1 was significantly better than those of clusters 2 and 3 (vs. clusters 2 and 3: *p* < 0.001). The score of cluster 2 was significantly better than that of cluster 3 (*p* = 0.046). The TMT Part A score of cluster 1 was significantly better than those of clusters 2 and 3 (H = 26.80, *p* < 0.001, vs. clusters 2 and 3: *p* < 0.001). However, cluster 2 was not significantly different compared with cluster 3 (*p* = 0.216).

**Figure 1:**
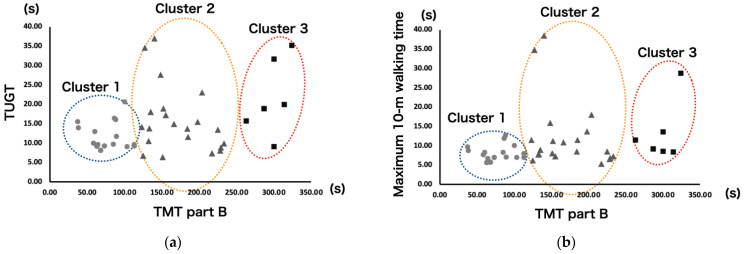
(**a**) Illustration of the Trail Making Test Part B and Timed Up and Go Test. Gray dots indicate cluster 1. Dark gray dots indicate cluster 2. Black dots indicate cluster 3. Cluster 1 showed better TUGT score than cluster 3 (*p* = 0.002). Cluster 2 showed better TUGT score than cluster 3 (*p* = 0.016). (**b**) Illustration of the Trail Making Test Part B and maximum 10 m walking speed test. Gray dots indicate cluster 1. Dark gray dots indicate cluster 2. Black dots indicate cluster 3. Cluster 1 showed better 10 m walking than cluster 3 (*p* = 0.024).

The maximum 10 m walking time and TUGT score of cluster 1 were significantly better than those of cluster 3 (maximum 10 m walking time: *p* = 0.024, TUGT: *p* = 0.005, Figure 1a,b). FIM motor, cognitive, and total scores were significantly higher in cluster 1 than those in cluster 2 (motor: *p* = 0.012, cognitive: *p* < 0.001, total: *p* = 0.004). However, there were no significant differences in MMSE, BRS, and sensory function of SIAS and BBS (*p* > 0.05, Table 2).

The results of the correlation analysis revealed that the TMT Part B was significantly positively correlated with the TMT Part A (ρ = 0.857, *p* < 0.001), TUGT (ρ = 0.300, *p* < 0.001), and maximum 10 m walking time (ρ = 0.290, *p* = 0.039). Moreover, the TMT Part B was significantly negatively correlated with the BBS (ρ = −0.390, *p* = 0.005), FIM motor, cognitive, and total scores (ρ = −0.350, *p* = 0.012, ρ = −0.564, *p* < 0.001, ρ = −0.422, *p* = 0.002, respectively).

## 4. Discussion

This study investigated the relationship between the degree of executive dysfunction and walking ability in patients with stroke. The participants were categorized into three groups according to executive dysfunction based on cluster analysis, as follows: the mild executive function group (cluster 1), the moderate executive function group (cluster 2), and the severe executive function group (cluster 3). Cluster 1 individuals were younger and had better walking ability than cluster 3 individuals, and ADL function was better in cluster 1 individuals than in cluster 2. The TMT Part B scores of cluster 2 individuals were significantly different from those of cluster 1 and cluster 3, but the walking ability was better than that of cluster 3 despite no significant difference from cluster 1. Cluster 3 individuals were older and had lower walking function than those in cluster 1. In addition, the study findings showed that executive function (the TMT Part B) was correlated with the maximum 10 m walking time, TUGT, BBS, TMT Part A, and FIM score. This study, therefore, indicated that the degree of executive dysfunction was associated with walking ability in patients with stroke.

Cluster 1 individuals were younger and had better walking and ADL function. Cluster 3 individuals were older and had lower walking function than those in cluster 1. This study supports the findings reported in previous studies [15,27,28]. Previous studies have observed that better executive function was associated with better walking ability in patients with stroke [15,27,28]. Hayes et al. investigated whether various executive dysfunctions were associated with the 10 m waking time in patients with stroke [15]. Their results indicated that better executive function was associated with a better 10 m walking time. In addition, the 10 m walking time was associated with the TUGT in patients with stroke [22], and the TUGT was related to executive function [27]. Heyes et al. investigated the relationship between executive function and physical function using a narrative review [27]. They found that executive function was associated with physical function. However, these studies should be considered because of the small number of subjects. Moreover, executive function was associated with ADL function and age [6,29,30,31]. Therefore, in patients with stroke, individuals in cluster 1 exhibited better walking and ADL function and were younger.

Notably, the walking ability of cluster 2 individuals was better than that of cluster 3 individuals but not significantly different from that of cluster 1. Moreover, cluster 2 exhibited lower ADL function than cluster 1. ADL function has been reported to be associated with executive function [6,30,31]. Pohjasvaara et al. investigated whether executive dysfunction was associated with ADL and physical function [30]. Their results implied that the present executive dysfunction group exhibited lower ADL function than the not-present executive dysfunction group despite the lack of significant differences in physical function between the two groups. Even with high physical function, attention and executive function are required for daily living. Therefore, cluster 2 had better walking ability despite lower ADL function.

In addition, executive function (the TMT Part B) was correlated with the maximum 10 m walking time, TUGT, BBS, TMT Part A, and FIM score. Our study supports the findings reported in previous studies [5,6,15,20,30,31]. Hayes et al. documented that the BADS can be used to classify groups into those with and without executive dysfunction and investigate differences in balance ability. Their findings showed that among patients with stroke, those without executive dysfunction had higher balance ability (BBS) [5]. Therefore, executive dysfunction was associated with balance ability.

This study has several limitations. First, the sample size was small. Second, given the cross-sectional nature of this study, the causal relationship between executive function and walking and balance ability could not be determined. Further longitudinal studies are therefore necessary. Third, this study included patients with ambulatory mild stroke. Therefore, there was a ceiling effect in BBS. Other assessments (i.e., the Balance Evaluation Systems Test) should be used in future studies. Finally, executive function was assessed using the TMT Part B. Therefore, only one limited aspect of executive function was assessed. Nonetheless, we believe that our use of the TMT Part B to classify executive function with cluster analysis provided accurate results, suggesting the presence of differences in physical function according to the executive function assessment.

## 5. Conclusions

In conclusion, this study may indicate differences in walking ability according to executive dysfunction in patients with stroke.

## Figures and Tables

**Table 1:** Characteristics of overall participants and the three clusters.

Variables	Overall (*n* = 51)	Cluster 1 (*n* = 20)	Cluster 2 (*n* = 24)	Cluster 3 (*n* = 7)	*p* Value
Age (years)	67.1 ± 13.3	60.7 ± 10.2	69.8 ± 10.8	76.0 ± 20.8	1 vs. 2: *p* = 0.056, 1 vs. 3: *p* = 0.020 *, 2 vs. 3: *p* = 0.724
Sex (men/female)	36/15	14/6	19/5	3/4	*p* = 0.178
BMI (kg/m^2^)	23.1 ± 3.2	22.9 ± 3.2	23.4 ± 3.0	23.0 ± 3.8	1 vs. 2, 1 vs. 3, 2 vs. 3: *p* = 1.000
Type of stroke (infarction/hemorrhagic)	30/21	13/7	13/11	3/4	*p* = 0.764
Paretic side (right/left)	15/36	3/17	10/14	2/5	*p* = 0.154
Time since stroke (day)	66.7 ± 46.0	60.0 ± 50.6	60.9 ± 41.3	105.9 ± 29.5	1 vs. 2: *p* = 1.000, 1 vs. 3: *p* = 0.046 *, 2 vs. 3: *p* = 0.075

Data are expressed as mean ± standard deviation. BMI: Body Mass Index. * *p* < 0.05 (Bonferroni method adjusted).

**Table 2:** Results of executive function and physical function in all participants and the three clusters.

Assessments	Overall (*n* = 51)	Cluster 1 (*n* = 20)	Cluster 2 (*n* = 24)	Cluster 3 (*n* = 7)	*p* Value
TMT-A (s)	71.8 ± 34.7	43.6 ± 16.2	82.0 ± 26.9	117.6 ± 30.6	1 vs. 2, 1 vs. 3: *p* < 0.001 *, 2 vs. 3: *p* = 0.216
TMT-B (s)	151.5 ± 78.9	77.8 ± 23.0	170.7 ± 38.7	296.4 ± 20.0	1 vs. 2, 1 vs. 3: *p* < 0.001 *, 2 vs. 3: *p* = 0.046 *
MMSE (points)	27.8 ± 2.2	28.5 ± 1.8	27.5 ± 2.0	26.7 ± 3.7	1 vs. 2: *p* = 0.297, 1 vs. 3: *p* = 0.798, 2 vs. 3: *p* = 1.000
TUGT (s)	15.67 ± 9.1	11.4 ± 3.4	15.6 ± 8.2	21.4 ± 9.1	1 vs. 2: *p* = 0.395, 1 vs. 3: *p* = 0.015 *, 2 vs. 3: *p* = 0.216
Maximum 10 m walking time (s)	21.4 ± 9.1	7.9 ± 2.0	11.5 ± 8.4	12.8 ± 7.3	1 vs. 2: *p* = 0.249, 1 vs. 3: *p* = 0.024 *, 2 vs. 3: *p* = 0.406
BRS upper-limb	5 (1–6)	5 (2–6)	5 (1–6)	6 (2–6)	1 vs. 2, 1 vs. 3, 2 vs. 3: *p* = 1.000
BRS finger	5 (1–6)	5 (2–6)	5 (1–6)	6 (2- 6)	1 vs. 2, 1 vs. 3, 2 vs. 3: *p* = 1.000
BRS lower-limb	6 (1–6)	6 (3–6)	5.5 (1–6)	6 (3–6)	1 vs. 2, 1 vs. 3, 2 vs. 3: *p* = 1.000
SIAS tactile sense	3 (0–3)	3 (1–3)	3 (0–3)	3 (2–3)	1 vs. 2, 1 vs. 3, 2 vs. 3: *p* = 1.000
SIAS position sense	3 (1–3)	3 (1–3)	3 (1–3)	2 (1–3)	1 vs. 2: *p* = 0.389, 1 vs. 3: *p* = 0.098, 2 vs. 3: *p* = 1.000
BBS (points)	49.0 ± 5.6	51.3 ± 4.9	48.3 ± 5.3	44.7 ± 6.2	1 vs. 2: *p* = 0.639, 1 vs. 3: *p* = 0.052, 2 vs. 3: *p* = 0.275
FIM motor (points)	68.5 ± 16.8	75.3 ± 15.5	61.2 ± 16.5	74.0 ± 11.7	1 vs. 2: *p* = 0.012 *, 1 vs. 3: *p* = 0.297, 2 vs. 3: *p* = 1.000
FIM cognitive (points)	28.5 ± 5.6	31.5 ± 5.7	26.0 ± 4.8	28.7 ± 4.2	1 vs. 2: *p* < 0.001 *, 1 vs. 3: *p* = 0.841, 2 vs. 3: *p* = 0.291
FIM total (points)	97.0 ± 21.4	106.8 ± 20.1	87.1 ± 20.3	102.7 ± 15.1	1 vs. 2: *p* = 0.004 *, 1 vs. 3: *p* = 0.427, 2 vs. 3: *p* = 1.000

Data are expressed as mean ± standard deviation or median (min–max). TMT: Trail Making Test, MMSE: Mini-Mental State Examination, TUGT: Timed Up and Go Test, BRS: Brunnstrom Recovery Stage, SIAS: Stroke Impairment Assessment Set, BBS: Berg Balance Scale, FIM: Functional Independence Measure. * *p* < 0.05 (Bonferroni method adjusted).

## Data Availability

Data are available from the corresponding author upon request.
